# Experimental articular cartilage repair in the Göttingen minipig: the influence of multiple defects per knee

**DOI:** 10.1186/s40634-015-0031-3

**Published:** 2015-06-18

**Authors:** Bjørn Borsøe Christensen, Casper Bindzus Foldager, Morten Lykke Olesen, Louise Vingtoft, Jan Hendrik Duedal Rölfing, Steffen Ringgaard, Martin Lind

**Affiliations:** Orthopedic Research Laboratory, Aarhus University Hospital, Nørrebrogade 44, building 1A, 1. Floor, Aarhus, Denmark; MR Research Center, Aarhus University Hospital, Skejby, Denmark; Department of Sports Traumatology, Department of orthopedic surgery, Aarhus University Hospital, Aarhus, Denmark

**Keywords:** Animal model, Articular cartilage, Göttingen minipig, Bone graft, Cartilage chips

## Abstract

**Background:**

A gold standard treatment for articular cartilage injuries is yet to be found, and a cost-effective and predictable large animal model is needed to bridge the gap between *in vitro* studies and clinical studies.

Ideally, the animal model should allow for testing of clinically relevant treatments and the biological response should be reproducible and comparable to humans. This allows for a reliable translation of results to clinical studies.This study aimed at verifying the Göttingen minipig as a pre-clinical model for articular cartilage repair by testing existing clinical cartilage repair techniques and evaluating the use of two defects per knee.

**Methods:**

Sixteen fully mature Göttingen minipigs were used. The minipigs received bilateral trochlear osteochondral drill-hole defects or chondral defects (Ø 6 mm), either one defect per knee or two defects per knee. The defects were treated with one of the following: Matrix-induced autologous chondrocyte implantation (MACI), microfracture (MFx), autologous-dual-tissue transplantation (ADTT), autologous bone graft, autologous cartilage chips. Empty chondral and osteochondral defects were used as controls. MRI and CT were performed 3 and 6 month, histology was performed 6 month postoperative.

**Results:**

The repair tissue varied in morphology from non-cartilaginous fibrous tissue to fibrocartilaginous tissue as seen on MRI, CT and histology at 6 month. The worst results were seen in the empty controls, while the best results were achieved with the MACI and ADTT treatment. The use of two defects per knee did not have any significant effect on the repair response.

**Conclusion:**

The outcomes of the applied treatments were consistent with the outcomes in clinical studies and it was possible to apply two defects per knee. The Göttingen minipig model was easy to handle, cost-effective and provided predictable outcome. Based on this study the use of two defects per knee, one in the medial and one in the lateral trochlear facet, in male Göttingen minipigs is recommended.

## Background

Articular cartilage does not heal spontaneously, and so far no treatment method has been able to regenerate hyaline cartilage in a consistent and predictable manner (Bae et al. 2006; Knutsen et al. 2007; Bentley et al. 2012; Gobbi et al. 2013; Bekkers et al. 2013; Christensen et al. 2015a). Several well established cartilage repair methods are available including microfracture (MFx) and matrix-induced autologous chondrocyte implantation (MACI). MFx results in short-term clinical improvements, but due to the fibrocartilaginous and fibrous nature of the repair tissue, the improvements tend to deteriorate after 2–5 years (Kreuz et al. 2006; Gobbi et al. 2013). MACI results in similar clinical improvements, and the results are longer lasting than with MFx (Behrens et al. 2006; Ventura et al. 2012), but the MACI treatment is limited by the need for two separate surgeries and the high cost of cell culturing. Recently, the use of cartilage chips is emerging as a potential treatment method. Studies have been published on particulated juvenile cartilage, and autologous cartilage chips in combination with scaffolds or embedded in fibrin glue (Cole et al. 2011; Farr et al. 2011; Marmotti et al. 2013; Farr et al. 2014; Christensen et al. 2015b), but long-term results are pending. To develop new surgical treatment methods and to test existing methods, cost-effective, reliable, and predictable large animal models are needed.

The Göttingen mini-pig has previously been used for cartilage repair research. Skeletal maturity is reached at the age of 18 months, defined by closing of the distal femoral growth plates and the characteristics of mature cartilage (Hurtig et al. 2011). Defects with a diameter of 5 mm has been shown to be critically sized (Gotterbarm et al. 2008). Skeletal maturity is important in order to prevent overestimation of repair response due to endogenous healing. The size of the animal, the joint size and the docile nature of the animal make the Göttingen minipig an appealing animal model in cartilage research. Nevertheless, possibility of application of clinical treatments and their repair responses has not been characterized sufficiently. Healing responses in the animal model as evaluated by histology and imaging should be comparable to those found in humans to allow for reliable translation of results from large animal studies to the clinical setting. Furthermore, the use of multiple cartilage defects per animal is important, both economically and ethically, since the utilization of multiple defects will allow the researcher to reduce the number of animals needed. However, the influence of multiple cartilage defects in one joint on cartilage repair response has not been investigated.

The present study aimed to 1) Validate the Göttingen minipig as a pre-clinical model for chondral and osteochondral repair using existing clinical cartilage repair techniques, and 2) Investigate whether or not two defects per knee (double-defect knees) will affect the repair outcome compared to single-defect knees.

We hypothesized that clinically available cartilage repair techniques could be applied in the Göttingen minipig and that the repair response would be similar to that found in clinical trials. Furthermore, we hypothesized that the repair outcome would not be affected by doubling the number of defects per knee, thereby reducing the number of Göttingen minipigs needed.

## Methods

### Study design

**Fig. 1:**
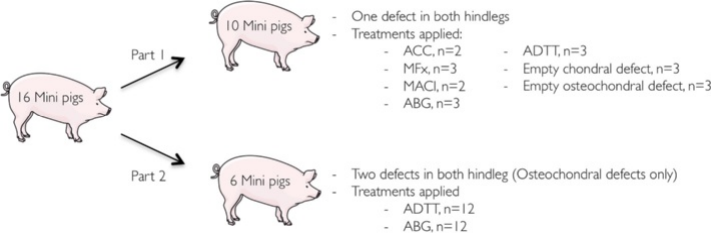
The study design. *ACC*, Autologous cartilage chips. *MFx*, Microfracture. *MACI*, Matrix induced autologous chondrocyte implantation. *ABG*, Autologous boe graft. *ADTT*, Autologous Dual-Tissue Transplantation. Pig sketch from www.servier.com

The study design is outlined in Fig. 1. The hypotheses were tested in two separate studies. All animals involved underwent bilateral knee surgery. One study focused on one defect per knee. Ten Göttingen minipigs were used (5 male, 5 female). The applicability and repair response of five different treatment methods and two different empty controls were tested in one defect per knee. The second study focused on the use of two defects per knee. Six Göttingen minipigs (6 male) received two defects in each knee. The outcome was compared between the neighboring defects, and between single-defect knees and double-defect knees.

The average age of the Göttingen minipigs was 21.0 months (18.5–23.7 months). The average weight was 41.9 kg (32.4–47 kg). The study complied with the Danish Law on Animal Experimentation and was approved by the Danish Ministry of Justice Ethical Committee, J.nr. 2012-15-2934-00301.

### Anesthesia

The animals were pre-medicated with Azaperone (Stresnil, 0.1 mL/kg, Janssen Pharmaceutica, Belgium) and Midazolam (Dormicum, 0.1 mL/kg, Hoffmann-La Roche AG, Switzerland) administrated subdermally. By an intravenous access was gained through the auricular vein general anesthesia was established with etomidate (Hypnomidate, 0.25 mL/kg, Janssen Pharmaceutica, Belgium), and a standard tracheal tube, size 6.5, was inserted. General anesthesia was maintained with Sevoflurane® (3 %, AbbVie, Denmark) and fentanyl (0.175 mL/kg/h, Hameln pharmaceuticals, Germany). Prior to the surgery, the animal was treated with prophylactic antibiotics (Penicillinprokain, Ceva Sante Animale, France) 0.03 mL/kg).

### Surgery

Local analgesics were administrated to the skin and periarticular tissue (Xylocain, 10 mL, 20 mg/mL, Astra Zeneca, Denmark). The location of the distal patella pole and the tibial tuberosity was marked on the skin, and a 5 cm midline skin incision directly over the patella ligament was made. The patella ligament was exposed, and access to the knee joint was gained by a trans-patella-ligamental incision from the distal patella pole to the tibial tuberosity. Through this access the intercondylar notch and trochlea was exposed.

In the single-defect knees, the Göttingen minipigs received chondral or osteochondral defects in the medial facet of the trochlear groove approximately two cm proximal to the intercondylar notch. In the double-defects knees, one osteochondral defect was made in the medial facet of the trochlear grove, approximately two cm proximal to the intercondylar notch, while the other osteochondral defect was made in the lateral facet of the trochlear grove, approximately 1 cm proximal to the medial defect. The chondral defects were outlined using a 6-mm skin biopsy punch and the cartilage was carefully stripped off using a curette until all cartilage including the calcified cartilage layer was removed, without any subchondral bleeding observed. Osteochondral defects 6 mm in diameter and 8 mm in depth were created using a cannulated 6 mm drill bit, which was preceded by drilling with a 2 mm guide wire. Low speed manual drilling was performed carefully to avoid heat damage to the tissue. After treatment was applied (Table 1) the patella ligament, subcutaneous tissue and skin were sutured. The animals were treated postoperatively with Finadyne 5 % (Flunixin meglumin, 1.1 mg/kg, oral paste, Intervet, Denmark) for five days, and were allowed free cage activity immediately after the operation.

**Table 1:** Description of the five different treatment groups and the empty chondral- and osteochondral control groups

Treatment	Type	*n*	Details
Empty full-thickness chondral defect	Full thickness chondral	3	Ø 6 mm full thickness chondral defect left untreated.
Autologous cartilage chips (ACC)	Full thickness chondral	2	This treatment was applied to a full-thickness chondral defect. A 6 mm chondral defect was created using a skin biopsy punch, and the harvested cartilage was cut into chips approximately 0.5 mm^2^, using a scalpel. The cartilage chips were embedded in fibrin glue and added to the defect, secured using fibrin glue.
Microfracture (MFx)	Full thickness chondral	3	The 6 mm defect was debrided so only the subchondral bony plate was left. Small perforations of the subchondral bone plate were made (Ø 1 mm), and the mesenchymal stem cells from the bone marrow cells were allowed to enter the full-thickness chondral defect.
Matrix-induced articular cartilage implantation (MACI)	Full thickness chondral	2	A collagen I/III membrane seeded with autologous chondrocytes was placed in a full-thickness chondral defect and secured using suture. Cartilage biopsies were performed one month prior to the surgery. The cartilage biopsies were cultured as previously described (Christensen *et al*. [2012]; Hansen *et al*. [2013]), and seeded onto the collagen I/III membranes one week prior to the surgery.
Empty osteochondral defect	Osteochondral	3	Ø 6 mm, 8 mm deep defect left untreated.
Autologous bone graft (ABG)	Osteochondral	15	A drill hole was made using a hand drill to avoid heat damage. The bone was collected and morselized into a paste and press-fitted into the osteochondral defect. Finally, the defect was sealed using fibrin glue (Tissel Duo Quick, Baxter Denmark).
Autologous Dual-Tissue Transplantation (ADTT)	Osteochondral	15	As above ABG is press-fitted into the osteochondral defect. A cartilage biopsy, taken before drilling, was cut into cartilage chips approximately 0.5 mm^2^ in size. The autologous cartilage chips (ACC) were then embedded in fibrin glue, and added onto the ABG

The defects of the single-defect knees were randomized into one of seven groups presented in Table 1. The double-defect knees were randomized to either autologous bone graft (ABG) or Autologous Dual-Tissue Transplantation (ADTT).

### Magnetic resonance imaging (MRI)

To evaluate the repair tissue surface, the subchondral tissue and the repair tissue signal intensity, each Göttingen minipig was MRI scanned prior to surgery, and three and six month postoperative using a 3 T whole-body MRI scanner (Magnetom Skyra; Siemens, Erlangen, Germany). The following sequences were used: Double Echo Steady State (DESS, 3D imaging with field-of-view (FOV) 151 × 180 × 90 mm; acquisition matrix 216 × 256 × 128; TR 13.5; TE 5.0; flip angle 28°; acquisition time 5 min 25 s), SPACE (3D imaging with FOV 98 × 180 × 78 mm; acquisition matrix 140 × 256 × 112; TR 1000; TE 40.0; ETL 52; acquisition time 3 min 57 s) and MPRAGE (3D imaging with FOV 120 × 183 × 99.8 mm; acquisition matrix 168 × 256 × 128; TR 2200; TE 2.65; TI 900; acquisition time 3 min 49 s).

For all the 3D imaging sequences an isotropic voxel of 0.7 × 0.7 × 0.7 mm^3^ was used.

OsiriX v. 5.8.5 64-bit (Pixmeo SARL, Bernex, Switzerland) was used for the analyses. An experienced, independent radiologist evaluated the images.

### Computed tomography (CT)

To evaluate the bone repair of the osteochondral lesion, repair tissue structure and presence of subchondral bone cysts, each animal was CT scanned three and six months postoperative using a SOMATOM Definition CT scanner (Siemens AG Medical Solutions, Erlangen, Germany). A clinical CT scanner was chosen over a Micro-CT due to its clinical relevance. In order to achieve the best-technically possible isotropic resolution of 0.6 × 0.6 × 0.6 mm^3^ the following scanning parameters were applied: 120 kV, 200 mAs, slice thickness 0.6 mm, spiral pitch factor 0.5 mm and reconstructed with a field of view of 307 mm^2^, an increment of 0.3 mm and filters of either B40f or B80f. The images were analyzed using OsiriX v. 5.8.5 64-bit (Pixmeo SARL, Bernex, Switzerland). To determine the bone defect volume in cm^3^, a region of interest (ROI) corresponding to the bone defect was drawn onto each 2D slide, and using the Osirix ROI volume tool the bone defect volume was computed. The bone defect repair percentage was calculated by comparing the remaining defect volume with the original drill hole defect. An experienced, independent radiologist evaluated the images.

### Histology

The animals were euthanized 6 months postoperative and the gross appearances of the defects were photographed. The samples were dehydrated in increasing concentrations of ethanol (70–96 %) at 4 °C. Clearing in 2-isopropanol and xylene preceded infiltration with methyl methacrylate (MMA) at 4 °C. The final polymerization of MMA was accelerated with N,N-dimethyl-p-toluidine at −20 °C. The samples were cut into 10 μm serial sections from the center of the defect using a microtome (Reichert Jung polycot).

Before staining the sections were deplasticized in dimetoxyethylacetate and rehydrated. The samples were stained with hematoxylin and eosin (HE), safranin-O, and toluidine blue.

In the first study the sample size of each treatment group allowed for evaluation according to cell morphology, glycosaminoglycan (GAG) staining and repair tissue surface. In the second study, blinded evaluation of the repair response was performed by two experienced staff members using the ICRS II scoring system (Mainil-Varlet et al. 2010).

### Animal welfare

Trained animal keepers, supervised by a veterinarian, closely observed each animal thrice daily. If the animal keepers assessed the Göttingen minipig to be limping or to be unwell, the animal was further evaluated according to general appearance, appetite, clinical findings (temperature, stool evaluation) and behavior, both when alone and when interacting with the animal keeper, each category was assigned a score of 1–4. Slight limping and decreased appetite and activity level was accepted for the first four days post-operative.

### Statistics

**Fig. 2:**
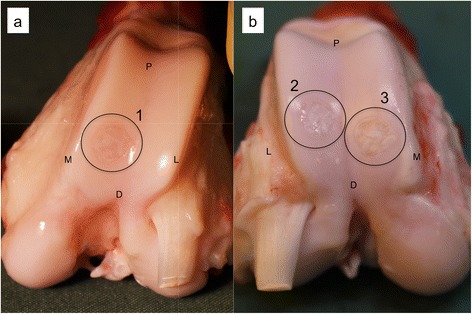
Macroscopic images of (**a**) a single defect knee, and (**b**) a double defect knee. The defects have been marked with circles 1–3. The defects in these images have all been treated with ADTT. Remains of cartilage chips can be seen in all three defects as white areas in the defect. *P*, Proximal, *D*, Distal, *M*, Medial and *L*, Lateral

Residuals were checked for normal distribution using QQ-plots. Bone defect fill from CT scans was evaluated using two-way ANOVA (time*treatment) with repeated measures (Prism 6, GraphPad Software, Inc., La Jolla, CA, USA). No interaction between the independent variables was observed and their effects were measured independently. Part 1: Due to high risk of a type 1 error, statistical tests were not used to investigate a difference between treatment methods. Part 2: All defects treated with ADTT and autologous bone graft were ICRS II scored. The treatments were grouped according to whether the defect was from a single-defect knee (Fig. 2, circle 1), if the defect was the proximal defect of double-defect knees (Fig. 2, circle 2) or if the defect was the distal defect of double-defect knees (Fig. 2, circle 3).

As the groups differed in size, and non-normal data distribution was confirmed using QQ-plots, Kruskal-Wallis one-way analysis of variance was used to test for a difference in repair response between the neighboring defects in the double-defect knees, and between the defects in single-defect knees and the defects in double-defect knees. For post-hoc evaluation, Dunn’s test was used. P-values less than 0.05 were considered significant.

## Results

No animals were limping, had reduced appetite nor reduced activity level after the initial five days of analgesic treatment. The trans-patella-ligament surgical approach provided adequate access to both the medial and lateral trochlear facet. Both the medial and the lateral trochlear facet were well suited for 6 mm defects.

**Fig. 3:**
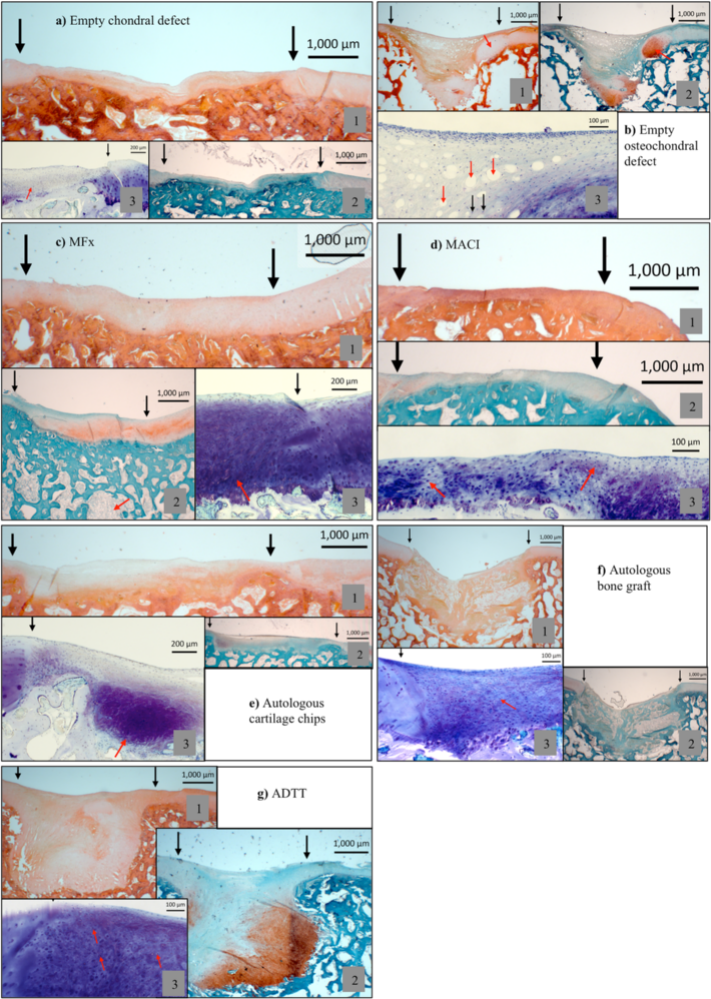
Histological images of the seven groups. In each group (**1**) is HE stained, (**2**) is Safranin-O stained and (**3**) is a higher magnification stained with Toluidine blue. **a1**-**3** Black arrows: Defect area. Red arrow: fibrous tissue. **b1** Black arrows: Defect area. Red arrow: An example of cartilage flow phenomenon. **b2** Black arrows: Defect area. **b3** Red arrows: vascular tissue, Black arrows: fibrocytes. **c1** Black arrows: Defect area, **c2** Black arrows: Defect area, red arrow: MFx drill tunnel. **c3** Black arrow: Transition area from healthy tissue to repair tissue. Red arrow: Fibrocartilage. **d1**-**3** Black arrows: Defect area. Red arrows: Chondrocytes in lacunaes embedded in fibrous tissue. **d1**-**3** Black arrows: Defect area. Red arrow: Cartilage chip. **f1**-**3** Black arrows: Defect area. red arrow: fibrocytes. **g1**-**3** Black arrows: Defect area, red arrows: Chondrocytes in lacunae embedded in fibrous tissue

**Fig. 4:**
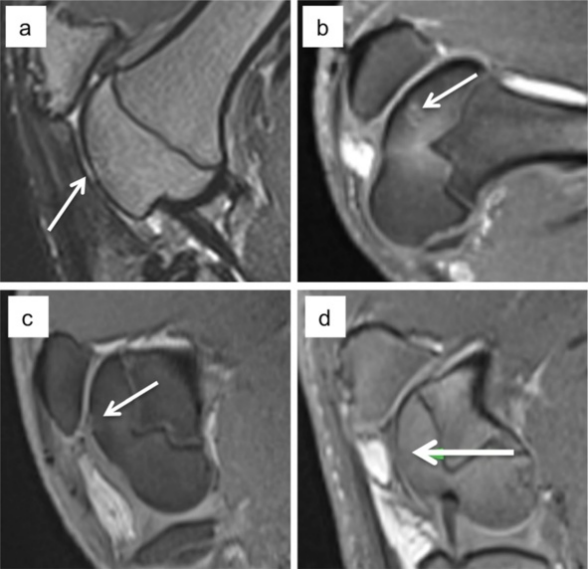
MRI of the four chondral treatment groups. **a** Empty chondral defect, **b** Microfracture. The MFx drill holes can be seen in the subchondral area. **c** Autologous cartilage chips and **d** MACI. The white arrows mark the defects

**Fig. 5:**
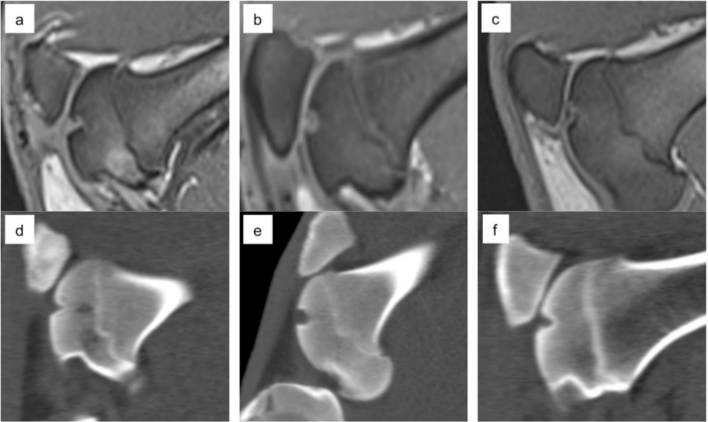
MRI and corresponding CT for the empty osteochondral defects (**a** and **d**), autologous bone graft (**b** and **e**) and the ADTT treated defects (**c** and **f**). Incomplete subchondral bone repair with a cyst-like appearance is seen in d)

Part 1: The histological results are presented in Table 2 and Fig. 3 and the radiological results are presented in Table 3, Figs. 4–5. Complete regeneration of hyaline cartilage was not observed in any treatment group.

**Table 2:** The histological results of all treatment groups, assessed according to cellular morphology, GAG staining and repair tissue surface

Treatment	Cellular Morphology	GAG-Staining	Repair tissue surface
Empty chondral defect (Fig. 3a)	Mixture of *fibrous* tissue and *fibrocartilage*	Negative	Smooth, but depressed
Autologous cartilage chips (Fig. 3e)	Predominantly *fibrocartilage*. Areas with *hyaline*-*like* tissue dispersed throughout the defect area	Negative	Smooth
MFx (Fig. 3c)	*Fibrocartilage*	<50 %	Smooth, but depressed
MACI (Fig. 3d)	H*yaline* cartilage in the periphery, *fibrocartilage* in the center	Negative	Smooth, but depressed
Empty osteochondral defect (Fig. 3b)	Predominantly *fibrous* tissue. Fibrocartilage present profoundly. Rich vascularity	<10 %	Smooth, but depressed
Autologous bone graft (Fig. 3f)	Mixture of *fibrous* tissue and *fibrocartilage*. Rich vascularity	<50 %	Smooth, but depressed. Slight fissuring observed
ADTT (Fig. 3g)	*Hyaline cartilage and fibrocartilaginous* tissue profoundly. *Fibrocartilage* and *fibrous* tissue superficially	<50 %	Smooth, but slightly depressed

*GAG*, Glycosaminoglycan. GAG staining percentage was assessed semi-quantitatively. *MFx*, Microfracture, *MACI*, Matrix-induced autologous chondrocyte implantation, *ADTT*, Autologous dual-tissue transplantation

**Table 3:** The radiological results of all treatment groups

Treatment method	Time-point	Bone defect repair (CT)	Repair tissue surface (MRI)	Subchondral tissue – MRI and CT
Empty chondral defect, (Fig. 4a)	3 months	NA	Persistent full-diameter chondral defect	Slight subchondral signal changes
	6 months	NA	Small persistent chondral defect, with surface fissuring	Slight subchondral signal changes
Autologous cartilage chips, (Fig. 4c)	3 months	NA	Intact surface, slightly irregular, with slight signal changes.	No subchondral signal changes
	6 months	NA	Intact surface, with slight signal changes.	No subchondral signal changes
MFx, (Fig. 4b)	3 months	NA	Slight surface depression, and surface fissuring. Repair tissue non-cartilaginous, non-bony	Signal changes around the MFx holes, and signs of subchondral cyst formation
	6 months	NA	Slight surface depression. No surface fissuring. Repair tissue non-cartilaginous, non-bony	Marked subchondral signal changes. No subchondral cyst formation
MACI, (Fig. 4d)	3 months	NA	Slight surface depression, but smooth surface covered with non-cartilaginous, non-bony tissue	Subchondral signal changes
	6 months	NA	Slight surface depression, but smooth surface covered with non-cartilaginous, non-bony tissue	No subchondral signal changes
Empty Osteochondral defect, (Fig. 5a, d)	3 months	50 %	Persistent defect	Persistent bone defect. The surrounding bone were less dense than native subcortical bone
	6 months	67 %	Marked surface depression with non-cartilaginous, non-bony tissue	Persistent bone defect. Sclerotic edge, and subchondral bone cyst formation
Autologous bone graft, (Fig. 5b, e)	3 months	63 %	Slight surface depression. Repair tissue non-cartilaginous, non-bony	Persistent bone defect, with fibrous repair tissue. No cyst formation or edema
	6 months	76 %	Slight surface depression. Repair tissue non-cartilaginous, non-bony	Persistent bone defect with fibrous repair tissue. No cyst formation or edema
ADTT, (Fig. 5c, f)	3 months	83 %	Slight surface depression. Defect partially covered by cartilage-like tissue	Superficial fibrous tissue, with profound bone repair. Small persistent bone defect
	6 months	92 %	Slight surface depression. Defect almost completely covered with cartilage-like tissue	Almost complete bone repair

The repair tissue surface was assessed using MRI, while the subchondral tissue was assessed using MRI and CT. The bone defect repair percentage is a measure of the degree of repair compared to the original defect volume. *MFx*, Microfracture, *MACI*, Matrix-induced autologous chondrocyte implantation, *ADTT*, Autologous dual002Dtissue transplantation

The *chondral* treatment groups all had a smooth, but depressed repair tissue surface, and very little GAG-positive staining. The worst results were found in the empty defects (Fig. 3a) where the tissue was predominantly fibrous, whereas the best results were found in the MACI group with a mixture of hyaline tissue and fibrocartilage (Fig. 3d). The defects were clearly distinguishable on MRI three and six months postoperative with a persistent large defect in the empty defects and smooth and slightly depressed surface of non-hyaline tissue in the MACI group (Fig. 4).

In the *osteochondral* treatment groups the worst results were also found in the empty defects, where fibrous tissue was predominant (Fig. 3b). The best osteochondral repair results were found in the ADTT group where a combination of hyaline tissue and fibrocartilage was predominant (Fig. 3g). MRI showed a marked surface depression in the empty defects while defect filling in the ADTT group was almost complete with repair tissue resembling healthy cartilage. CT imaging showed a significant increase in bone volume from 3 to 6 month (p = 0.033) indicating continuous subchondral bone regeneration after 3 months follow-up. The average increase in subchondral bone volume from 3 to 6 month was 36 % and the average bone deficit after 6 month was only 0.06 cm^3^ (SD ± 0.04) compared with the native subchondral bone level.

Part 2: In the ADTT and ABG treated knees there were no significant difference in ICRS II score between the neighboring defects nor were there any significant difference between the neighboring defects and the single-defect knees in any subcategory. The results of each ICRS II subcategory can be seen in Table 4.

**Table 4:** The ICRS II subcategories for the single defect knees and the double defect knees of study 2

ICRS II category	ADTT proximal	ADTT distal	ADTT single	ABG proximal	ABG distal	ABG single
Tissue morphology	38.3 ± 8.2	41.7 ± 7.3	36.7 ± 1.7	15.8 ± 6.4	20.0 ± 7.7	11.7 ± 3.3
Material staining	67.3 ± 11.2	70.0 ± 11.3	86.6 ± 8.8	40.0 ± 10	51.7 ± 15.5	35.0 ± 27.8
Cell morphology	40.0 ± 7.2	40.0 ± 8.2	36.7 ± 1.7	16.7 ± 6.3	20.8 ± 7.7	11.7 ± 3.3
Clusters	55.8 ± 14.5	67.6 ± 17.5	90.0 ± 5.7	69.2 ± 12.7	57.5 ± 19	58.3 ± 26.8
Surface architecture	35.0 ± 6.7	38.3 ± 11.1	36.7 ± 18.6	64.2 ± 10.4	40.1 ± 5.5	23.3 ± 13.7
Basal integration	60.0 ± 5.2	52.5 ± 8.7	40.0 ± 0	64.2 ± 5.2	54.2 ± 10.7	25.0 ± 7.6
Tidemark formation	24.2 ± 10.8	20.8 ± 9.3	21.7 ± 8.3	8.3 ± 3.3	19.2 ± 11.6	8.3 ± 4.4
Subchondral bone	23.3 ± 9.4	25.8 ± 9.6	35.0 ± 7.6	45.8 ± 6.9	49.2 ± 10.5	18.3 ± 7.3
Vascularization	58.3 ± 18.5	65.0 ± 14.3	40.0 ± 23.1	35.8 ± 15.1	49.2 ± 21.3	33.3 ± 33
Surface assessment	30.8 ± 7.9	26.7 ± 9.1	31.7 ± 15.9	35.0 ± 11.4	34.2 ± 9.2	21.7 ± 14.8
Deep assessment	47.5 ± 7.3	43.3 ± 7.6	43.3 ± 12	46.7 ± 8.3	58.3 ± 6	18.3 ± 4.4
Overall assessment	48.8 ± 3	50.0 ± 3.3	52.0 ± 3	48.1 ± 3.4	49.4 ± 4.4	31.0 ± 3.7

“Single”, “proximal” and “distal” corresponds to Fig. 2, circle 1, 2 and 3 respectively. No significant difference was found between any defect location in neither the ADTT group nor the ABG group

## Discussion

The primary findings of the present study was that the histological and radiological outcomes of clinically applied chondral and osteochondral repair techniques exhibited similar healing characteristics as seen in previous studies on the Göttingen minipig and in clinical situations. Furthermore, we found no change in outcome having two defects in per knee joint as compared to one, thus advocating the use of two defects in future studies.

The aim of surgical treatment of cartilage injuries in humans is the relief of symptoms and the return to normal daily activities and sports. Inherently these parameters cannot directly be measured in animal models, and a successful outcome is ultimately based on the biological healing response evaluated by histology and radiology. In a recent study on Yukatan minipigs, Fisher *et al*. found that untreated full-thickness chondral defects were incompletely filled with fibrous and fibrocartilaginous tissue 6 weeks post-operative (Fisher et al. 2015). Similar results have been reported by Gotterbarm *et al*. who investigated the histological outcome of untreated chondral- and osteochondral defects in the Göttingen minipig. The authors evaluated empty chondral and osteochondral defects from 62 Göttingen minipigs. They found that empty chondral defects were filled mainly with fibrous and partially fibrocartilaginous tissue. The empty osteochondral defects were concavely depressed and filled with fibrous tissue and fibrocartilage with blood vessels present in the defect and signs of the cartilage flow phenomenon (Gotterbarm et al. 2008). These results were mirrored in the untreated chondral- and osteochondral defects of the present study as seen in Table 2 and Fig. 3a and b. However, chondral and osteochondral repair techniques were not tested in the study by Gotterbaum *et al*..

In a clinical situation MFx will often result in a significant short-term clinical improvement with deterioration of long-term outcome. The repair tissue morphology, as seen in the present study as well, is fibrous or at best fibrocartilaginous (Gobbi et al. 2005; Saris et al. 2008; Vanlauwe et al. 2011; Gobbi et al. 2013). Both Zuo *et al*. and Fisher *et al*. studied MFx in adolescent minipigs and found that defects treated with MFx were filled with fibrocartilage with rounded cells in lacunae situated in disarranged fibrous matrix, as seen in the present study (Fig. 3c) (Zuo et al. 2007; Fisher et al. 2015). These findings are comparable to clinical histological outcome as seen by Gudas *et al*., who found that 57 % of MFx repair tissue was fibrocartilaginous, while 43 % was fibrous (Gudas et al. 2005a). Karthikyan *et al*., found the MFx repair tissue to be fibrocartilaginous (Karthikeyan et al. 2012) while Bae *et al*., found a mixture of fibrocartilage and hyaline-like tissue (Bae et al. 2006).

In the present study MACI produced predominantly hyaline tissue and fibrocartilaginous repair tissue (Fig. 3d). This is also well established in clinical studies. Zheng *et al*., reported 75 % hyaline-like cartilage 6 months after surgery, and Bartlett *et al*. reported 36 % of the repair tissue to be hyaline-like, the remainder being fibrous (Bartlett et al. 2005; Zheng et al. 2007). Anders *et al*. found 33 % to be a mixture of hyaline-like tissue and fibrous tissue, with 41 % being fibrocartilaginous (Anders et al. 2008).

One of the main responsibilities of researchers performing animal experiments is to minimize the suffering of the animal by considering: 1) *Reducing* the number of animals used. 2) *Refining* the surgical technique and the care and housing facilities and 3) *Replacing* the animal studies with *in vitro* studies when possible. This was introduced by Russell *et al*. in 1959 and is known as “the three R’s” (Russell and Burch 1959). In the present study we established that doubling the number of defects per knee did not affect the repair outcome or cause post-operative mortality. This enables researchers to achieve the same number of defects while halving the number of animals used. This reduces the cost of the studies and addresses ethical concerns, however one must remember that the biological variation is reduced in the process.

Several animal models are available for articular cartilage research. As described in the above, the biological repair response must resemble what is seen in a clinical situation, but the size of the animal, the cartilage thickness, and ethical concerns must also be considered before choosing a suitable model. Small animal models as the rabbit are frequently used in articular cartilage research, but the model suffers from a high level of endogenous repair making the model best suited for proof-of-concept studies rather than clinical translation (Chu et al. 2010). The dog, sheep and goat models are roughly the same size as the minipig, and all have been used in cartilage repair studies. The articular cartilage of the dog and minipig shares the same collagen arrangement as in humans (Kaab et al. 1998). Furthermore, dogs, unlike rabbits, goats, sheep and minipigs, can be trained to accept braces to limit weight bearing, to further mimic the clinical situation. While the dogs’ status as a family pet complicates its use from an ethical standpoint, the fact that goats and sheep are ruminant animals limits the available housing and surgical facilities due to the risk of prion disease transmission. The horse model has an articular cartilage thickness of 1.5–2 mm which is significantly closer to humans (2–2.5 mm) than rabbits (<0.3 mm), sheep (0.4–0.5 mm), dogs and minipigs (0.5–0.8 mm) or goats (0.9–1.5 mm) (Shortkroff et al. 1996; Hunziker 2002; Frisbie et al. 2006; Gotterbarm et al. 2008). This makes the horse model an obvious choice if human-like cartilage thickness is required, however, the acquisition cost and handling difficulties significantly limits a more widespread use.

The Göttingen minipig is close, but not equal, to humans regarding weight, joint size and articular cartilage collagen arrangement. The docile nature of the minipig, and the fact that a stable weight of 30–50 kg is maintained in adulthood makes handling the animal very easy. Furthermore, the blood count, blood clotting parameters, electrolytes and liver enzymes have similar values to those found in humans (Marshall et al. 1972; Rispat et al. 1993). However, the Göttingen minipig is limited by the relatively thin articular cartilage, which complicates the use of radiological follow-up due to resolution limitations of MRI. Furthermore, the Göttingen minipig does not accept braces to limit postoperative weight bearing.

The present study is limited by the small sample size per treatment method. This prevents comparison of results between groups but allows for a larger number of treatments methods to be tested. The number of Göttingen minipig knees assigned to each treatment group did not allow for statistical analysis between treatment outcomes, however, the high consistency in outcome within each treatment group was an important finding since the outcomes reported are consistent with the results of current clinical studies. The use of both male and female pigs is an important study limitation. However, Rispat *et al*. showed that no biochemical or haematological differences between male and female Yucatan minipigs existed and to further minimize the risk of gender bias, the treatments were randomization. Still, the existence of a gender bias must be considered.

Additional surgical experience using the Göttingen minipig was achieved in a pilot study where a medial parapatellar approach was tested. This approach was more time demanding, caused greater surgical trauma and resulted in more postoperative pain as observed by limping on the operated leg in the animals. The parapatellar approach was therefore abandoned for the transpatella-ligament approach. Furthermore, the surgeons found that the male Göttingen minipig had a more slender hind leg, which improved surgical access to the trochlea compared with the female.

## Conclusion

Based on the results of this study, the authors recommend the Göttingen minipig as a suitable model for cartilage repair. At six months follow-up of clinically relevant treatment methods, histology was comparable to that found in humans. CT imaging proved crucial to the monitoring of subchondral bone repair, and is recommended in the evaluation of all osteochondral treatments. The use of two defects in each knee on male Göttingen minipigs proved to be the most cost-effective approach.

## Abbreviations

ACC: Autologous cartilage chips
MACI: Matrix-induced autologous chondrocyte implantation
MFx: Microfracture
ADTT: Autologous Dual-Tissue Transplantation
MRI: Magnetic resonance imaging
CT: Computed tomography
DESS: Double echo steady state
FOV: Field of view
ETL: Echo train length
SPACE: 3D turbo spin echo with variable flip angle
TE: Echo time
TR: Repetition time
MMA: Methyl methacrylate
HE: Hematoxylin and eosin
BSA: Albumin bovine serum
PBS: Phosphate buffered saline
GAG: Glycosamino glycane
